# Collagen and elastic fibres in acute and chronic liver injury

**DOI:** 10.1038/s41598-021-93566-1

**Published:** 2021-07-15

**Authors:** Andrew Hall, Corina Cotoi, Tu Vinh Luong, Jennifer Watkins, Prithi Bhathal, Alberto Quaglia

**Affiliations:** 1 Sheila Sherlock Liver Centre, Royal Free London NHS Foundation Trust, London, UK; 2 Department of Cellular Pathology, Royal Free London NHS Foundation Trust, London, UK; 3 Department of Pathology, University of Melbourne, Parkville, VIC Australia; 4 Department of Cellular Pathology, Royal Free Hospital and UCL Cancer Institute, London, UK

**Keywords:** Liver diseases, Liver cirrhosis, Liver fibrosis, Medical research

## Abstract

The histological distinction between acute and chronic liver injury is a challenging aspect of liver histopathology. It is traditionally based on the interpretation of morphological changes to the extracellular matrix (ECM) at sites of hepatocyte loss using histochemical stains. Our aim was to investigate whether immunohistochemistry and multiplexing for collagen type (I & III) and elastic fibres and a modified Victoria blue method could be helpful. We studied 43 livers removed at transplantation for acute liver failure (ALF, 20 cases) or cirrhosis (23) plus 8 normal controls. In ALF the periportal ECM was normal in 2 cases, contained mainly collagen I associated with a ductular reaction in 6 cases, and delicate elastic strands in 11 cases. Periportal deposition of mainly collagen I and mature elastic fibres was observed in cirrhosis. In ALF the perisinusoidal ECM was intact in 4 cases, collapsed or condensed but of normal composition (predominantly collagen III) in 2 cases, and collapsed and condensed containing mostly collagen I in 17 cases (7 including delicate immature elastic strands). In contrast, bridging fibrous septa of cirrhosis contained abundant collagen 1 and bundles of mature elastin. We propose the use of a scale and the use of immunohistochemistry and multiplexing in additional to histochemical stains to characterise the ECM changes in acute and chronic liver injury.

## Introduction

A challenging aspect in liver pathology is the interpretation of changes occurring in areas of hepatocyte loss when differentiating between acute and chronic liver injury. This is often characterised by a distinction between an active profibrogenic deposition of connective tissue fibres and pre-existing connective tissue fibres collapsed and condensed from hepatocyte dropout^1^. Pan fibrillar collagen histochemical techniques such as picro-Sirius red (SR)^2,3^ and Van Gieson (VG)^4^ are helpful for this purpose, but there is considerable variation in collagen staining protocols and patterns that can influence histological interpretation^5^. Collagen content demonstrated by SR and VG consists principally of collagen I and III^6^ , which are best differentiated from each other using immunohistochemistry (IHC), preferentially in a dual epitope staining protocol.

Previous studies have found that the demonstration of elastic fibres within an area of liver injury can also help in assessing whether a lesion is recent or long standing^7,8^. Elastic fibres are composed of a sheath of elastin fibre micro-fibrillar protein (EFMP) binding several proteins that house a tropo-elastin core^9,10^. The proportional composition of EFMP and tropo-elastin monomer within elastic fibres allows the distinction of three categories of fibre type^10^. Mature elastin fibres contain both a crosslinked tropo-elastin monomer core and an EFMP sheath. Similarly, elaunin has both an elastin fibre core and micro-fibrillar sheath but the proportion of tropo-elastin monomer in elaunin is lower than in the more mature fibres. Oxytalan is composed solely of EFMP^11^.

The assessment of elastic fibres using resorcin fuchsin dyes such as Victoria Blue (VB)^12^, aldehyde fuchsin or orcein^10^ is usually part of the routine diagnostic work-up of liver specimens in cellular pathology laboratories. The oxidation step which is routinely part of these elastin staining protocols is thought to create acidic products from cystine oxidation that allows ionic links to the fuchsin dyes^13^. This allows the dyes to bind to both the EFMP and the elastin core. It is possible to differentiate between those with and without the tropo-elastin core, i.e. differentiate oxytalan from elaunin and mature elastin by omitting the oxidation step. This has also been found to equate well to the diameter of the fibre bundle and the microscopic objective at which it can be seen^14^.

Our aim was to describe the changes to the composition of the extracellular matrix (ECM) in terms of collagen types (I & III) using multiplex IHC and elastic fibres (Oxytalan vs. Elaunin and mature elastin) using VB and elastin IHC in areas of hepatocyte loss occurring in acute and chronic liver injury. We have studied a retrospective cohort of livers removed at transplantation for acute liver failure (ALF) or cirrhosis and a control group of histologically normal liver. We have selected conventional histochemical stains that are commonly used in this scenario and explored how they can be complemented by single and double epitope immunohistochemistry.

## Material and methods

Our retrospective study cohort consisted of 48 livers removed at transplantation for ALF (28) or cirrhosis (20). Twelve of the 20 cases of cirrhosis were due to alcohol-related injury and 8 were due to chronic hepatitis C infection. The background liver of 8 specimens derived from surgical resection of metastatic adenocarcinoma of colorectal origin with no significant histological abnormality were used as normal controls. Histological review was undertaken and hepatocellular necrosis was described as zonal (affecting part of the lobule), massive (pan-lobular multi-lobular hepatocyte necrosis with almost complete loss of hepatocytes) or submassive (areas of pan-lobular multi-lobular necrosis alternating with regenerative nodules), as described by Lefkowitch^1^.

From the routine H&E and SR stained sections, one paraffin block from each case was selected from an area representative of the overall pattern of injury. In the case of submassive necrosis the selected sample included preserved nodular parenchyma and areas of multilobular necrosis.

From each block 6 serial sections were cut. The sections were stained in the following sequential order with (i), Haematoxylin and Eosin, (ii) IHC anti-Elastin antibody (ab9519), (iii) VB with the oxidation step omitted (demonstrating only mature elastin fibres and elaunin), (iv) VB with oxidation step (demonstrating oxytalan in addition to elaunin and mature elastin), (v) IHC double epitope stain for collagen I (ab6308) and collagen III (ab7778); (vi) SR. The full protocols for the techniques are available in Supplementary Information.

The collagen I/III double epitope IHC were analysed using a Nuance multispectral imaging digital camera^15^. In brief, the Nuance camera takes multiple images at a series of wavelengths of light at 10 nm intervals. The resulting image data allows a more detailed spectral signature for each chromogen than with a conventional RGB camera. This allows us to more accurately spectrally unmix the chromogens from one another. The resultant images can be converted to a digital signal of a different colour allowing better visualisation of the two epitopes.

For a selection of cases that showed either extensive collagen I positivity or those with initial small amounts of collagen I around areas of ductular reaction we duplicated the stains using single antigen IHC to correlate and confirm that of the double antigen stain. In an additional single case, we used cytokeratin 19 (CK19) in an IHC double stain with elastin to better identify the biliary epithelium^16^ (Fig. 3). The protocols are also available in Supplementary Information.

The sections of each case were reviewed and analysed with particular attention to the changes affecting the periportal area and the areas of hepatocyte loss. These findings are presented in terms of the presence of a ductular reaction, alteration to the periportal and perisinusoidal ECM, including the type of collagen and the presence and composition of elastic fibres. Particular care was taken to differentiate true alteration of the ECM composition in areas of confluent collapse from ambient tangentially cut hepatic veins.

All work was covered under ethical approval 07/Q0501/50, HRA and HCRW by the Hampstead Research Ethics Committee in which due to the retrospective nature of the study the requirement for informed consent has been waived. All tissue was handled and protocols carried out by a state registered Health and Care Professional Council member. Tissue was assessed by fellows of the Royal College of Pathologists. All relevant guidelines and regulations were followed. No organs involved in the research were procured from prisoners.

## Results

All 6 staining methods were of appropriate high quality in all of the consecutive serial sections in 43 out of 56 cases. The double stain for collagen I and collagen III failed to reach standard in four cases and the elastin stain failed in one case, in likelihood due to defective fixation. Thus, our study cohort of cases of ALF was based on 12 cases of submassive necrosis, 6 cases of massive necrosis and 5 cases of confluent necrosis due to paracetamol (acetaminophen) overdose (POD) related injury. The details of the 23 cases with massive/submassive necrosis and POD related injury are shown in Table 1. Twenty cases of cirrhosis and 8 cases of near-normal liver completed our series.

**Table 1:** Details of the patients with livers showing massive/submassive necrosis and paracetamol overdose.

Pt	Age	Sex	Cause	Time interval (presentation to transplantation)	Type of injury	Periportal changes			Intralobular changes		
						Ductular reaction	Predominant collagen type	Elastic fibres	Perisinusoidal ECM	Predominant perisinusoidal collagen type	Perisinusoidal elastic fibers
1	20	F	POD	3 days reported	Panlobular	Present	III	None	Intact	III	None
2	26	F	POD	5 days	Zonal (centro-midlobular)	Present	III	None	Intact	III	None
3	38	F	POD	3 days	Zonal (centro-midlobular)	Present	III	None	Intact	III	None
4	40	M	POD	2 days	Zonal (centro-midlobular)	None	III	None	Intact	III	None
5	23	F	POD	17 days	Zonal (centro-midlobular)with nodular transformation	None	III	None	Collapsed and condensed	III mainly focal delicate I	None
6	28	M	HBV	10 days	MHN	Present	I	None	Collapsed	III mainly focal delicate I	None
7	34	M	Indeterminate	< 3 weeks	MHN	Present	I	None	Collapsed and condensed	III mainly focal delicate I	None
8	27	F	Indeterminate	1 week	MHN	Present	I	None	Collapsed and condensed	III mainly focal delicate I	None
9	20	F	Indeterminate	2 weeks	MHN	Present	III	None	Collapse	III	None
10	35	F	HBV	2 weeks	MHN	Present	I	None	Collapsed	III mainly focal delicate I	None
11	19	M	Indeterminate	DILI 2 months earlier. Improved. Sudden decline OLT after 8 days	MHN	Present	I	Delicate strands (oxytalan)	Collapsed and condensed	III plus I (dense)	None
12	50	M	Isoniazid	2 months	Submassive	Present	I	None	Collapsed and condensed	III plus I (dense)	None
13	51	M	Isoflurane	2 weeks	Submassive	Present	I	Delicate strands (oxytalan)	Collapsed and condensed	III plus I (dense)	None
14	54	F	Indeterminate	6 weeks	Submassive	Present	I	Delicate strands (oxytalan)	Collapsed and condensed	III plus I (dense)	Delicate strands (oxytalan
15	47	F	Indeterminate	6 months	Submassive	Present	I	Delicate strands (oxytalan)	Collapsed and condensed	III plus I (dense)	Delicate strands (oxytalan)
16	53	M	Indeterminate	2 weeks	Submassive	Present	I	None	Collapsed and condensed	III mainly focal delicate I	None
17	58	F	Indeterminate	7 weeks	Submassive	Present	I	Delicate strands (oxytalan)	Collapsed and condensed	III plus I (dense)	None
18	36	F	Khat	Several months	Submassive	Present	I	Delicate strands (oxytalan)	Collapsed and condensed	III plus I (dense)	None
19	32	F	Indeterminate	9 days	Submassive	Present	I	Delicate strands (oxytalan)	Collapsed and condensed	III	None
20	17	F	Indeterminate	6 weeks	Submassive	Present	I	Delicate strands (oxytalan)	Collapsed and condensed	III plus I (dense)	Delicate strands (oxytalan)
21	50	F	Indeterminate	5 months	Submassive	Present	I	Delicate strands (oxytalan)	Collapsed and condensed	III plus I (dense)	Delicate strands (oxytalan)
22	37	M	Indeterminate	10 months	Submassive	Present	I	Delicate strands (oxytalan)	Collapsed and condensed	III plus I (dense)	Delicate strands (oxytalan)
23	26	M	HBV	4 weeks	Submassive	Present	I	Delicate strands (oxytalan)	Collapsed and condensed	III plus I (dense)	Delicate strands (oxytalan)

HBV = Hepatitis B virus, POD = Paracetamol Overdose.

### Normal liver

**Figure 1:**
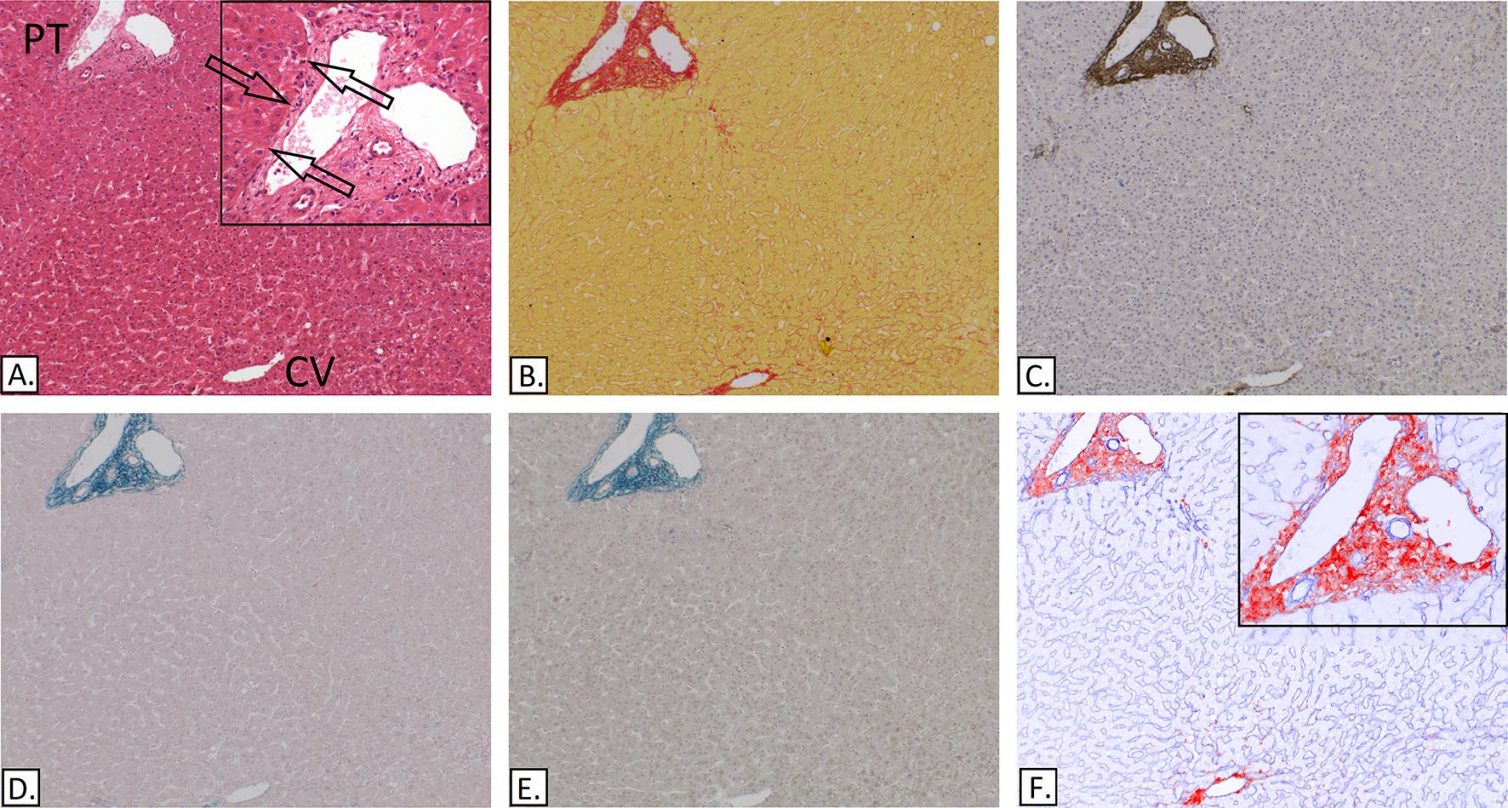
Demonstration of the 6 techniques used and their localization in normal liver showing the centrilobular hepatic venule (CV) to portal tract (PT) axis. The arrows mark the limiting plate. (**A**) H&E. (**B**) Picro-Sirius red. (**C**) IHC-Elastin, (ab9519). (**D**) Victoria Blue. (**E**) Modified Victoria blue—no oxidation step. (**F**) IHC-collagen I (ab6308) in red & collagen III (ab7778) in blue, spectrally unmixed channels from Nuance camera + software.

In normal livers (Fig. 1) the portal ECM was composed mainly of collagen I and mature elastic bundles with a sharp edge facing the limiting plate. The perisinusoidal ECM was made mainly of collagen III with no elastic fibres. Collagen I and mature elastic fibres marked the centrilobular hepatic venules in places.

### Minimal or no changes to the periportal-perisinusoidal ECM

**Figure 2:**
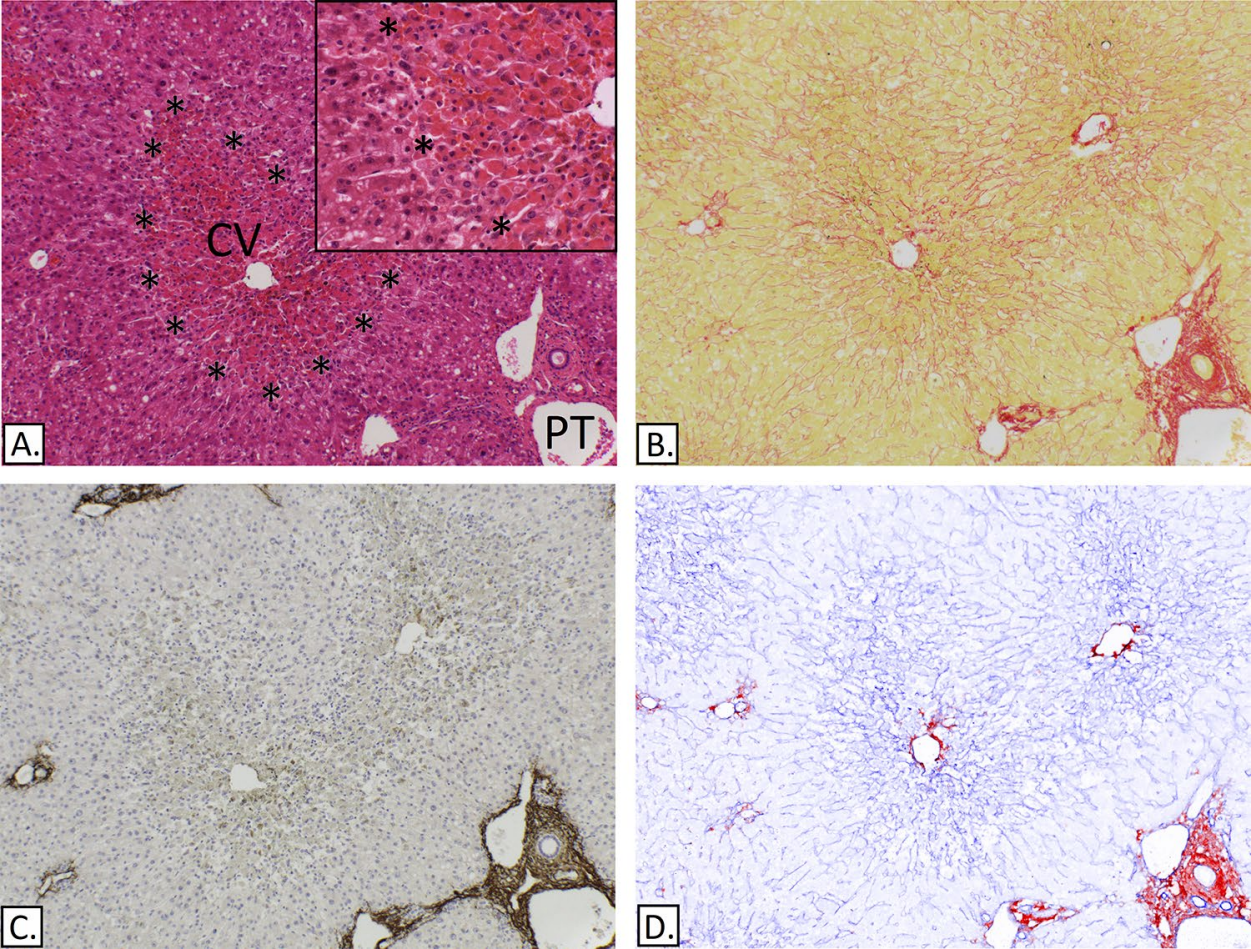
Liver from a patient with a paracetamol overdose. The images show confluent necrosis sparring peri-portal hepatocytes without collapse or change in composition of the perisinusoidal ECM. There is no ductular reaction. (**A**) H&E, confluent necrosis (demarcated by asterisk). (**B**) Picro-Sirius red. (**C**) IHC-Elastin, (ab9519). (**D**) IHC-collagen I (ab6308) in red & collagen III (ab7778) in blue, spectrally unmixed channels from Nuance camera + software. Collagen I and elastin fibre bundles are restricted to portal tracts (PT). And collagen I marks the centrilobular hepatic venules (CV).

**Figure 3:**
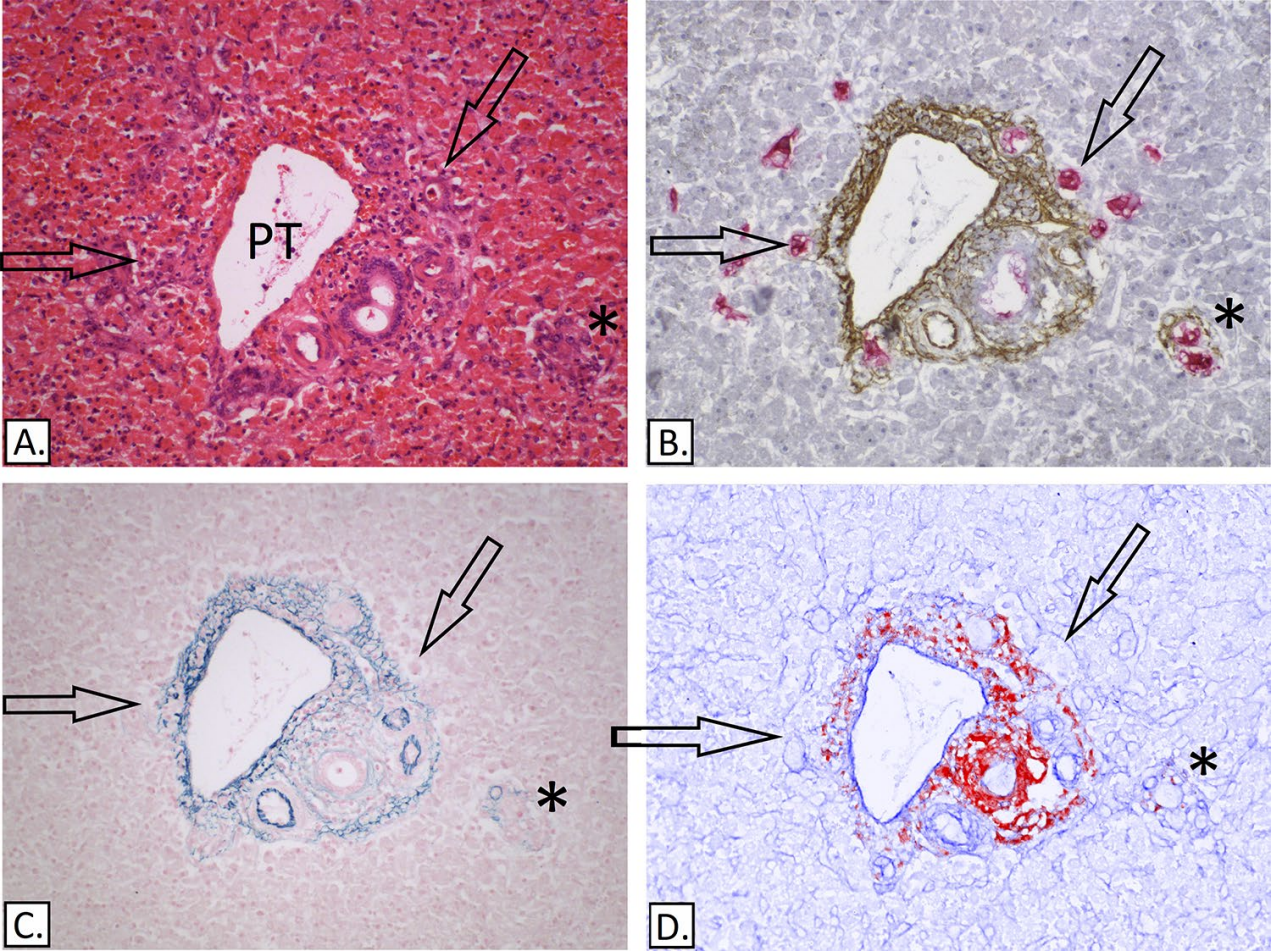
Liver from a patient with a paracetamol overdose. A portal tract (PT) occupies the central part of the field. (**A**) H&E. (**B**) IHC-Elastin DAB (ab9519) and cytokeratin 19 (CK19), (NCL-CK19) in red. (**C**) Victoria blue. (**D**) IHC-collagen I (ab6308) in red & collagen III (ab7778) in blue, spectrally unmixed channels from Nuance camera + software. Arrows identify peri-portal ductular epithelium confirmed by the expression of CK19. Collagen I fibre bundles are restricted to portal tracts. Asterisks show a tangentially cut portal branch.

**Figure 4:**
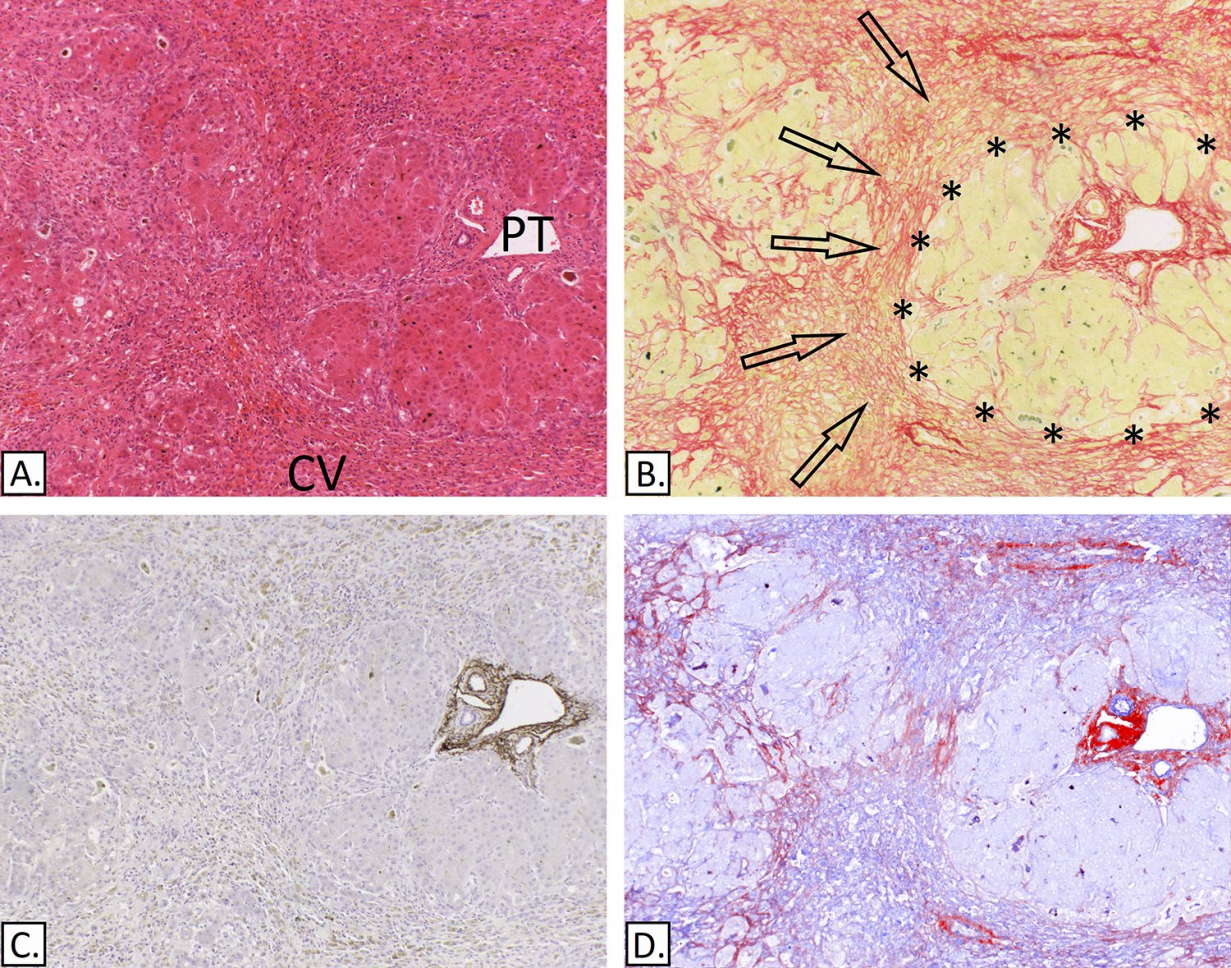
Liver from a patient with a paracetamol overdose. (**A**) H&E. The portal tract (PT) and centrilobular hepatic venule (CV) are labelled. (**B**) picro-Sirius red, arrows mark the mid-centrilobular ECM and asterisk the nodular configuration of the hepatocytes. (**C**) IHC-Elastin (ab9519). (**D**) IHC-collagen I (ab6308) in red & collagen III (ab7778) in blue, spectrally unmixed channels from Nuance camera + software. The hepatocytes show a somewhat nodular configuration suggesting an attempt at regeneration demarcated by the asterisks in B. There is condensation of the intervening ECM framework, predominantly collagen III (D.) marked by the arrows.

In 6 (1–5 and 9) of the 23 patients transplanted for ALF, the portal ECM appeared to be preserved and very similar in configuration to the portal ECM in the normal controls. Five of these 6 cases showed the typical hepatocellular injury of POD related toxicity, sparing periportal hepatocytes in four cases and involving the entire lobule in one. In the remaining case (patient 9) the parenchymal injury was in the form of massive necrosis. In 2 of the 3 cases with POD-related zonal necrosis that spared periportal hepatocytes there was no ductular reaction (Fig. 2). It was mild in the other POD-related cases and more florid in the case of massive hepatic necrosis. The ductular reaction appeared to be located in the periportal space immediately outside the original portal boundaries. The periportal ECM was made of mainly collagen type III. No elastic fibres were identified (Fig. 3). In four of the POD-related cases the perisinusoidal ECM in the areas of zonal necrosis appeared intact and composed of mainly collagen III. In the remaining POD case (Case 5, Fig. 4), the periportal hepatocytes showed a somewhat nodular configuration suggesting an attempt at regeneration, possibly related to a relatively prolonged time interval between injury and transplantation (17 days). In this case the mid-centrilobular ECM appeared condensed, staining strongly with SR. The collagen here was predominantly of type III with few delicate strands of collagen I identified in the most centrilobular aspect likely related to the hepatic venule. No elastic fibres were present in these areas. The collapsed ECM in the case of massive hepatic necrosis was composed predominantly of collagen III. No elastic fibres were identified.

### Deposition of collagen I in the periportal region and/or areas of hepatocyte loss

**Figure 5:**
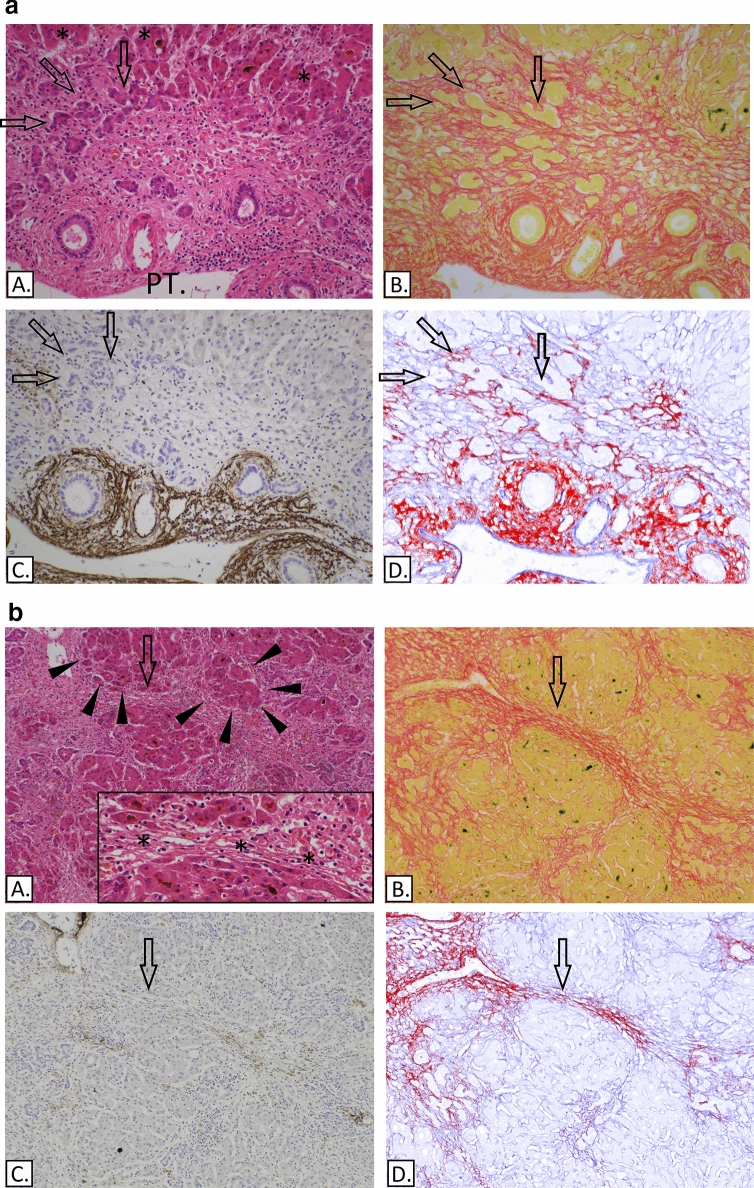
(**a**) Submassive hepatic necrosis. (**A**) H&E, a portal tract (PT) is shown and asterisks mark hepatocytes. (**B**) Picro-Sirius red. (**C**) IHC-Elastin (ab9519). (**D**) IHC-collagen I (ab6308) in red & collagen III (ab7778) in blue, spectrally unmixed channels from Nuance camera + software. Peri-portal ductular reaction (arrows) is associated with collagen I deposition (D) but there is no presence of elastic fibre bundles (C). (**b**) Submassive hepatic necrosis. (**A**) H&E, this image shows regenerative nodules made of hepatocytes (examples demarcated with triangles). The inset shows the confluent hepatocyte loss (asterisks), at higher magnification. (**B**) Picro-Sirius red. (**C**) IHC-Elastin (ab9519). (**D**) IHC-collagen I (ab6308) in red & collagen III (ab7778) in blue, spectrally unmixed channels from Nuance camera + software. The arrows indicate a septum corresponding to an area of confluent hepatocyte loss and condensation of the ECM that contains a predominance of collagen I type fibres (D) but no elastic fibres (C).

A periportal ductular reaction was present in another 6 of the 23 livers of patients transplanted for ALF (patients 6, 7, 8, 10, 12, 16). In these six cases the ductules rested on delicate strands including collagen I but with no identifiable elastic fibres (Fig. 5a). In 4 of these cases (patients 6, 7, 8 and 10) the parenchymal injury was massive necrosis. In the other 2 cases (patients 12 and 16) necrosis was submassive and preserved hepatocytes were arranged in small nodules. In all 6 cases the perisinusoidal ECM had collapsed and in 4 cases it had condensed to form denser septa (patients 7, 8, 12 and 16). The collapsed ECM was mainly composed of collagen III and an associated focal component of delicate strands of collagen I in 5 cases. More dense obvious strands of collagen I were present in patient 12 (Fig. 5b). No elastic fibres were identified.

### Deposition of delicate elastic fibres in the periportal region and/or areas of hepatocyte loss

**Figure 6:**
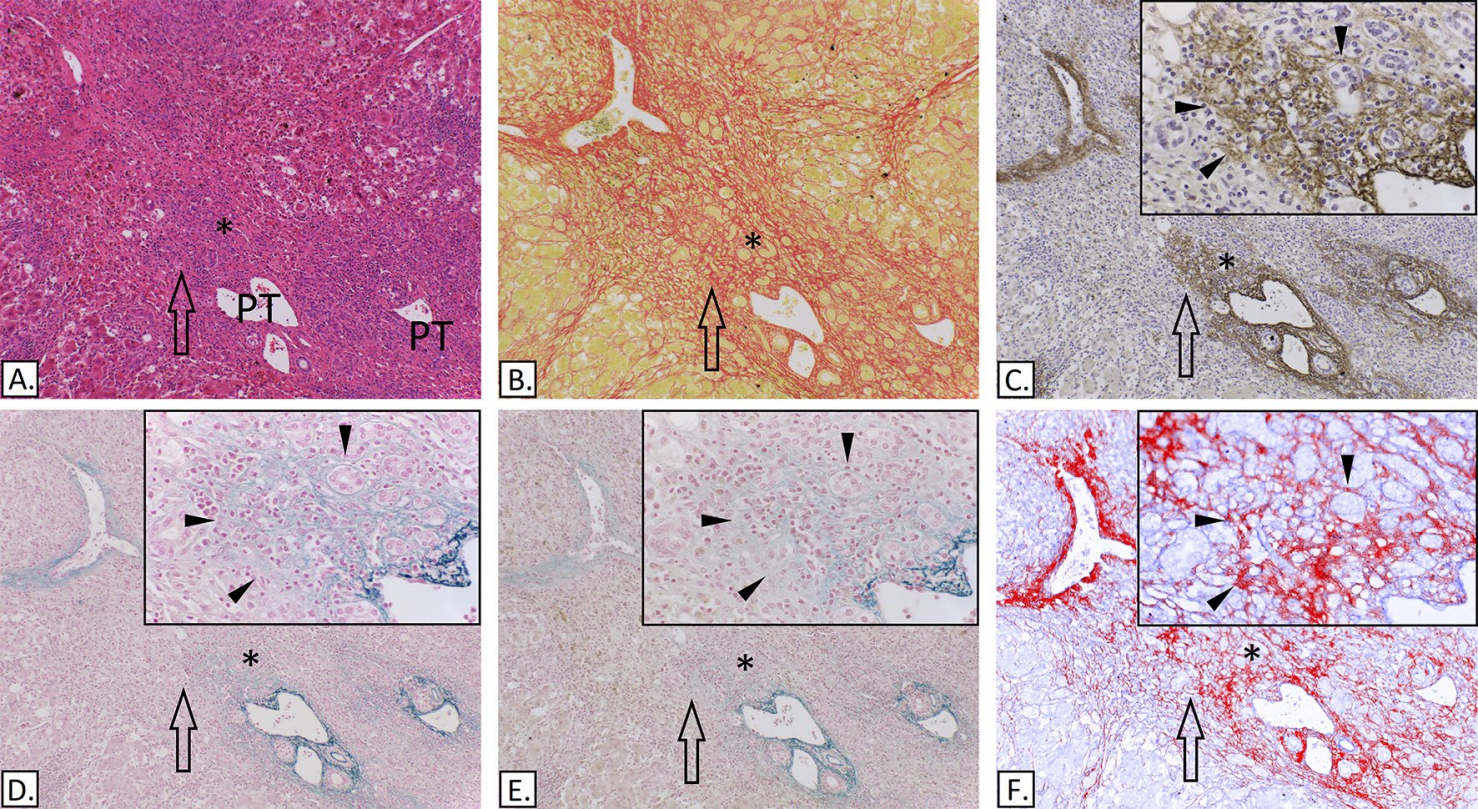
Submassive hepatic necrosis. (**A**) H&E. (**B**) Picro-Sirius red. (**C**) IHC-Elastin (ab9519). (**D**) Victoria Blue. (**E**) Modified Victoria blue—no oxidation step. (**F**) IHC-collagen I (ab6308) in red & collagen III (ab7778) in blue, spectrally unmixed channels from Nuance camera + software. The portal tracts (PT) are labelled, the asterisks show an area of ductular reaction and the arrows indicate the same periportal area for comparison of different stains. The insets are high-power images of the peri-portal ductular reaction the triangles are indicating the elastic fibres (C, D, E) and the collagen I (F). In this case the ductules are surrounded by collagen I (F) and there is delicate elastic fibre deposition (C & D), note the differential staining between the Victoria blue (D) and the modified method (E).

In 11 cases of ALF (patients 11, 13–15, 17–23) the periportal ductular reaction was characterised by ductules resting on a loose ECM composed of clearly visible collagen I and delicate strands of elastic fibres (Fig. 6). The difference in stain between the two Victoria blue staining methods (with and without oxidation steps) suggested the presence of oxytalan fibres in these cases.

**Figure 7:**
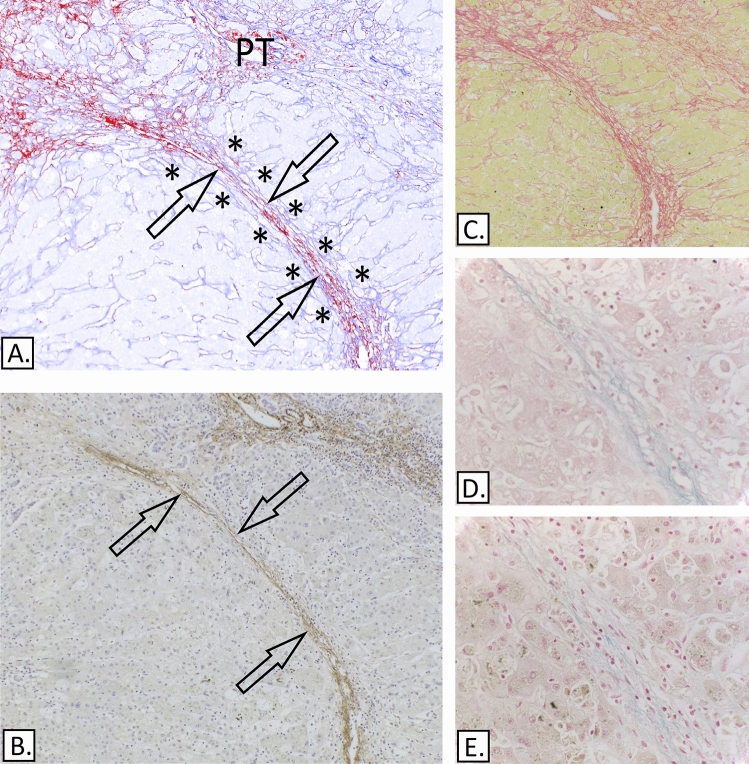
Submassive hepatic necrosis. (**A**) IHC-collagen I (ab6308) in red & collagen III (ab7778), spectrally unmixed channels from Nuance camera + software. It shows an area of parenchyma collapse (arrows) between two nodular areas (asterisks). (**B**) IHC-Elastin (ab9519). (**C**) Picro-Sirius red. (**D**) Victoria Blue. (**E**) Modified Victoria blue—no oxidation step. The collapsed area contains collagen I (A) and delicate elastin fibres (B), including oxytalan, as demonstrated by the differential staining between the standard Victoria blue (D) and the modified Victoria blue method (E).

Necrosis in most (10/11) of these cases was submassive. The collapsed perisinusoidal ECM, particularly in proximity of the parenchymal nodular areas, often formed dense septa and in 9 cases contained collagen I positive bundles as well as collagen III. Very delicate elastic strands (oxytalan) were present in 6 of these 10 cases (patients 14, 15, 20–23) and were of a similar appearance and composition to those present in the periportal region (Fig. 7).

In case 11 necrosis was massive and the collapsed and condensed perisinusoidal ECM contained collagen I but no elastic fibres.

### Strong deposition of collagen I and elastic fibre bundles in cirrhosis

**Figure 8:**
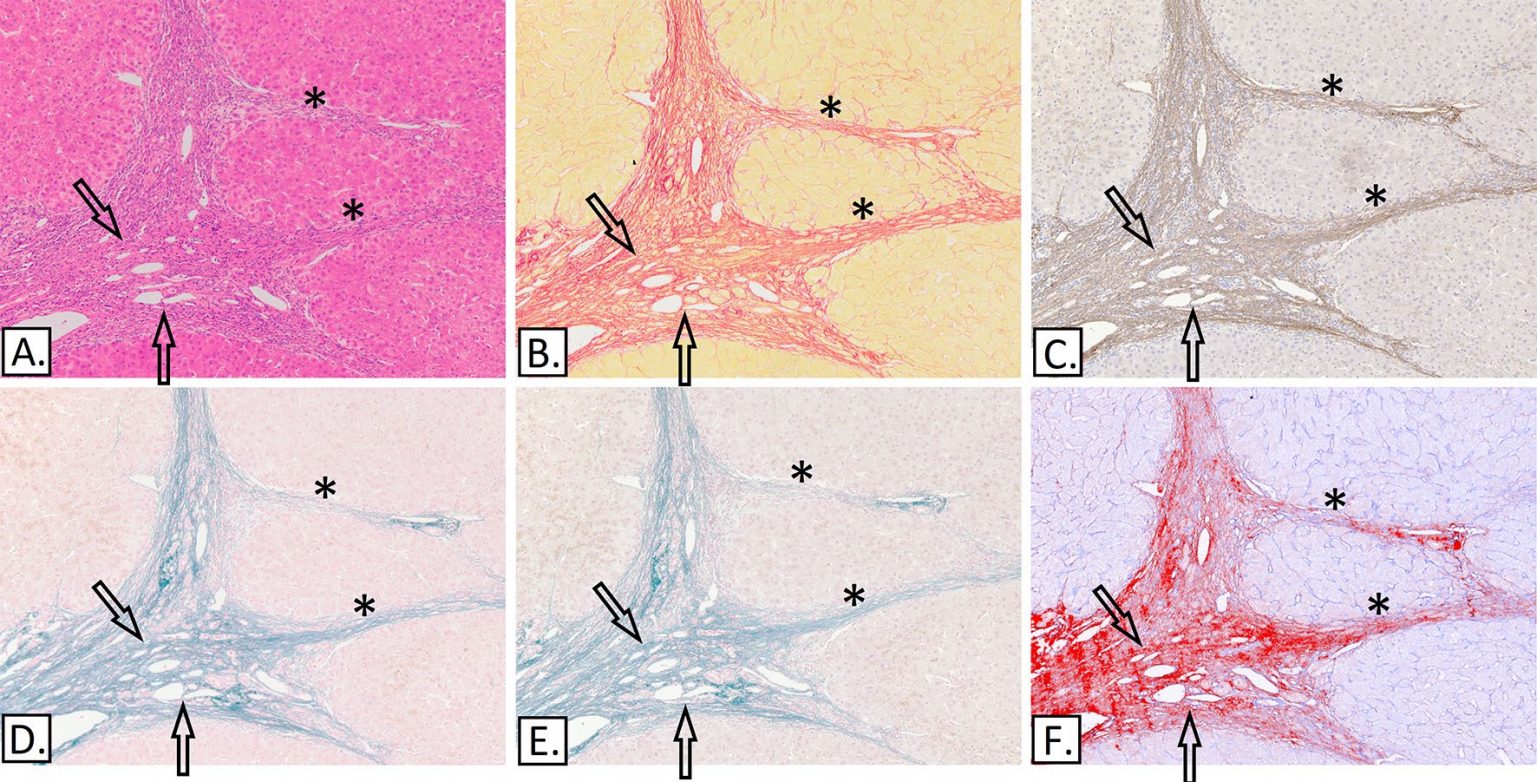
Alcohol related cirrhosis. (**A**) H&E. (**B**) Picro-Sirius red. (**C**) IHC-Elastin, (ab9519). (**D**) Victoria Blue. (**E**) Modified Victoria blue—no oxidation step. (**F**) IHC-Collagen I (ab6308) in red & collagen III (ab7778), spectrally unmixed channels from Nuance camera + software. Portal tract remnant (arrows) and fibrous septa (asterisks) containing both collagen I and mature elastic fibres as demonstrated by the lack of differential staining between the standard Victoria blue (D) and the modified Victoria blue method (E).

All 20 cases of cirrhosis were characterised by the presence of abundant collagen I and dense elastic bundles in and around the residual portal tracts and the fibrous septa. In some instances, with collagen I or elastin stains, it was possible to recognise the original portal tract profile and in other cases the septal ECM blended with the portal tract remnant. The elastic bundles stained strongly positive for elastin IHC and with both Victoria blue methods, i.e. there was little difference when the oxidation step was omitted indicating the presence of mature elastic fibres (Fig. 8). In some instances, slender, and at times incomplete septa were noted that were made of mature elastic fibres, collagen I and III, suggesting an element of regression.

## Discussion

The histological distinction between acute and chronic liver injury is a challenging aspect of liver histopathology. It is traditionally based on the interpretation of changes affecting the reticulin framework at sites of hepatocyte loss. The separation between passive (collapsed) and active (fibrotic) septa rests on their morphology, tinctorial properties (e.g. golden appearance of fibrotic septa when using an untoned Gordon and Sweet reticulin stain), the presence of collagen I inferred by the intensity (two-tone) and staining pattern when using trichrome staining methods^1,5^ and the identification of elastic fibres^7,8^.

Our work based on the combination of histochemical and immunohistochemical method, allowed us to investigate in detail the type of elastic and collagen fibres in the livers removed at transplantation from patients with acute or chronic liver injury. Our observations suggest that the changes to the ECM can be classified as follow:

### Minimal, early, reactive/adaptive

In some patients with zonal or massive necrosis the periportal ECM shows a normal composition. A ductular reaction, when present, is outside the well demarcated portal boundaries. The ECM in areas of zonal or panlobular hepatocyte loss is intact or collapsed but of normal, predominantly collagen III, composition. These changes were associated, in our series, with POD related injury and probably reflected the short time interval (few days to 1–2 weeks) between the injury and the precipitous clinical decline leading to transplantation.

### Collagen I deposition

Collagen I is identified around periportal ductules. The collapsed perisinusoidal ECM in areas of hepatocyte loss, consists of collagen III fibres stacked up to form relatively dense septa which at times include delicate strands of collagen I. These changes in our series were associated with massive necrosis or the formation of small parenchymal nodules suggesting the early stage of a regenerative process. The time interval between onset of symptoms and transplantation in our cases ranged from 1 week to 2 months. The increase ratio of collagen I to collagen III content in more damaged liver is corroborated by similar findings in tissue homogenates^6^.

### Early elastification

Delicate strands of elastic fibres including oxytalan overlay the collagen strands around periportal ductules. In the SR stained sections the portal-periportal boundary is blurred. It is still recognisable in the VB, elastin or collagen stained sections. These periportal changes, in our series, were associated with submassive necrosis characterised by large parenchymal nodules alternating with large areas of multilobular hepatocyte loss. The perisinusoidal ECM, particularly in between nodular hepatic plates, is collapsed, compressed, usually contains collagen, including visible collagen I strands and at times includes very delicate elastic strands of similar composition to the periportal ones (i.e. EFMP oxytalan rather than elaunin/mature elastin containing the tropo-elastin polymer).

These changes can potentially be difficult to distinguish from the ECM alterations in cirrhosis. The time interval between onset of symptoms and transplantation in our cases ranged from 4 weeks to 10 months. These more advanced ECM changes are likely the result of a dynamic balance between the nature, severity and progression rate of the parenchymal injury and a regenerative attempt. The liver of these patients may appear shrunken and nodular on cross sectional imaging and there may be signs of portal hypertension creating a clinical dilemma in terms of differentiating between acute injury, decompensation of chronic liver disease and acute on chronic liver injury^17^.

### Established advanced/end stage chronic liver disease

The deposition of dense bundles of collagen including abundant type I collagen and mature elastic fibres made principally of elastin characterise the cirrhotic stage. Bundles of collagen, abundant in collagen type I, and elastin are present around ductules in the proximity of portal remnants which at times cannot be discerned. Slender incomplete septa suggestive of regression were observed particularly in the livers from patients with alcohol-related liver injury and could be related possibly to the abstinence required prior to transplantation^14^.

The retrospective nature of our series is a limitation in terms of interpreting the precise chronology of the spectrum of changes we have described. Other limitations include sampling variation and the 2-dimensional nature of conventional histology in projecting changes to the complex hepatic 3-dimensional structure. Portal/periportal boundaries and the extent and distribution of confluent injury can be difficult to define, particularly in delineating zonal residues and differentiating vascular remnants from true bridging collapse. We also cannot entirely exclude that some of the ECM changes observed (e.g. perisinusoidal strands of collagen I) may be due to pre-existing liver injury, and that existing inflammatory activity can modify ambient connective tissue framework^14,18^. The immunoreactivity observed does not fully reflect the complex 3D structure of collagens in the hepatic ECM.

Our findings suggest that the changes affecting the ECM are part of a continuum and relate to the balance between a range of factors including the severity and pace of progression of the parenchymal injury, efficiency of regeneration and local as well as systemic inflammatory responses. The histological and clinical overlap between injury of recent onset and of long-standing activity also suggests that a strict dichotomist approach in classifying patients with acute or chronic liver injury works well with the extreme ends of the spectrum (e.g. POD related liver injury vs end stage alcohol-related cirrhosis) but less so in the middle. Fluctuations in disease activity, sampling variation and the practical constraints in monitoring tissue recovery by repeated serial liver biopsies also limit our understanding of the more or less natural course of some types of liver injury, set reversibility thresholds and prognosticate. With these considerations in mind we propose the adoption of a scale (Tables 2, 3) to describe histologically the intermediates between acute and chronic liver damage defined by ECM content. This scale separates the histological signs of tissue damage and recovery from strict and to some extent arbitrary chronological points (e.g. 6 months is habitually used as the boundary between acute and chronic liver injury), and can be used as a reference when correlating histological, radiological, and clinical findings or other tissue based exploratory techniques. We believe the use of immunohistochemistry in addition to the more conventional assessment of connective tissue components based on histochemical methods (including VB ± the oxidation step) can be informative. In our experience staining for collagen I, III and elastin is rewarding and allows us to better demonstrate some of the dynamics of connective tissue formation in liver fibrosis and may be complemented by other markers, e.g. fibrillin and fibulins^19,20^. This approach is made increasingly feasible by progress in multiplexing techniques allowing for optimisation of tissue sample usage and we found that the use of multispectral cameras and their unmixing algorithms improved our ability to interpret the panoply of visual data from such techniques. This proposed scale is intended as a basis that can be adapted and developed at later stages.

**Table 2:** Peri-portal stromal changes.

Scale	Periportal ECM changes (changes from previous point in scale in bold)
0	Normal portal tracts and periportal hepatic plates. (H&E, HC) No changes to periportal collagen. (IHC) Collagen I: predominant in portal tracts and hepatic venules in places. (IHC) Collagen III: portal tracts and perisinusoidal ECM. (IHC) Elastic fibres: restricted to portal tracts and hepatic venules in places. No elastic fibres in perisinusoidal ECM. (HC and IHC)
1	Normal portal tracts + /− inflammatory activity. No ductular reaction. (H&E, HC) No changes to periportal collagen. (IHC) No elastic fibre deposition. (HC and IHC)
2	**Periportal ductular reaction + /− inflammatory activity (H&E)** No changes to periportal collagen(IHC) No elastic fibre deposition (HC and IHC)
3	**Collagen I: fibres also present around ductular reaction** (IHC) No elastic fibre deposition (HC and IHC)
4	Collagen I: fibres also present around ductular reaction (IHC) **Elastin: weakly positive fibres around ductular reaction** (IHC) **VB: differentially stains with and without oxidation step; i.e. fine fibres around ductular reaction are only visible with VB that includes oxidation**
5	Collagen I: fibres also present around ductular reaction (IHC) **Elastin: strongly positive fibre bundles around ductular reaction**(IHC) **VB: fibre bundles present around ductular reaction regardless of the oxidation step** **Portal/periportal boundaries still recognisable**
6	Collagen I: fibres also present around ductular reaction (IHC) Elastin: strongly positive fibre bundles around ductular reaction VB: fibre bundles present around ductular reaction regardless of the oxidation step **Boundaries of portal tract remnant are blurred**

Please note that the difference between 0 and 1 on the scale is the presence of hepatocyte injury usually in the form of zonal or sometimes panlobular/multilobular hepatocyte loss.

H&E: Haematoxylin and eosin.

HC: Histochemical stains (e.g. reticulin, orcein, Victoria blue (VB), picrosirius red (SR).

IHC: immunohistochemical stains (e.g. collagen I, collagen III, elastin).

**Table 3:** Parenchymal ECM changes.

Scale	Parenchymal ECM changes (changes from previous point in scale in bold)
0	Normal hepatic plates No changes to periportal collagen Collagen I: predominant in portal tracts and hepatic venules in places Collagen III: portal tracts and perisinusoidal ECM Elastic fibres: restricted to portal tracts and hepatic venules in places. No elastic fibres in perisinusoidal ECM
1	Perisinusoidal ECM intact (HC) No change in collagen type (IHC) Elastic fibres not present (HC and IHC)
2	**Perisinusoidal ECM collapsed but not condensed (HC) with its delicate strands still discernible** No change in collagen type (IHC) Elastic fibres not present (HC and IHC)
3	**Bridging condensed septa (HC)** No change in collagen type (IHC) Elastic fibres not present (HC and IHC)
4	Bridging condensed septa (HC) **Collagen I: delicate fibres present (IHC)** Elastic fibres not present (HC and IHC)
5	Bridging condensed septa (HC) **Collagen I: coarse fibre bundles present (IHC)** Elastic fibres not present (HC and IHC)
6	Bridging condensed septa (HC) Collagen I: coarse fibre bundles present (IHC) **Weakly positive delicate elastic fibres in places (HC and IHC)** **VB: differentially stains with and without oxidation step, i.e. fine fibres are only visible with VB that includes oxidation**
7	Bridging condensed (broad or slender(regressive) septa (HC) Collagen I: coarse fibre bundles present (IHC) **IHC—Elastin: clearly positive fibre bundles** **VB: fibre bundles present regardless of the oxidation step**

Please note that the difference between 0 and 1 on the scale is the presence of hepatocyte injury usually in the form of zonal or sometimes panlobular/multilobular hepatocyte loss.

H&E: Haematoxylin and eosin.

HC: Histochemical stains (e.g. reticulin, orcein, Victoria blue (VB), picrosirius red (SR).

IHC: immunohistochemical stains (e.g. collagen I, collagen III, elastin).

## Supplementary Information

Supplementary Information.
