# Supplementary material for: Collagen and elastic fibres in acute and chronic liver injury
Source: Sci Rep. 2021 Jul 15;11:14569. doi: 10.1038/s41598-021-93566-1 (PMC8282791; doi:10.1038/s41598-021-93566-1)
Supplement: Supplementary file 1 — Supplementary Information. [file 41598_2021_93566_MOESM1_ESM.docx]

**Collagen and elastic fibres in acute and chronic liver injury.**

**Andrew Hall^1^, Corina Cotoi^2^, Tu Vinh Luong^2^, Jennifer Watkins^2^, Prithi Bhathal^3^ and Alberto Quaglia^4^.**

**^1^ Sheila Sherlock Liver Centre, Royal Free London NHS Foundation Trust**

**^2^Department of Cellular Pathology, Royal Free London NHS Foundation Trust**

**^3^Department of Pathology, University of Melbourne, Parkville, VIC, Australia.**

**^4^ Department of Cellular Pathology, Royal Free Hospital and UCL Cancer Institute.**

**Correspondence to: alberto.quaglia@nhs.net**

**Supplementary information.**

**Victoria blue protocol.**

1. Take FFPE sections through xylene and alcohol to distilled water.
2. Treat sections with acidified potassium permanganate for 5 minutes.
3. Wash sections in water and bleach with 1% aqueous oxalic acid until sections clear (approx. 1 min).
4. Wash sections in running tap water.
5. Wash sections in 70% aqueous alcohol.
6. Stain in Victoria blue solution, in a sealed coplin jar overnight, (approx. 16 hours).
7. Wash well in 70% aqueous alcohol.
8. Wash in running tap water for 1 minute.
9. Stain in nuclear fast red solution for 5 mins.
10. Wash in running tap water for 2 minutes
11. Dehydrate clear and mount.

Victoria blue solution:

100ml Distilled water

0.25 g Dextrin

2g Resorcinol

1g Victoria blue dye

1. Mix these reagents and gradually warm until boils whilst adding 12.5ml of boiling 29% aqueous ferric chloride and continue to boil for 3 mins.
2. Cool solution and filter (Whatman grade 1).
3. Dry the residue on the filter paper at room temp and recover the dried residue.
4. Dissolve the powder in 1% acid alcohol (1% HCL in 70 aqueous alcohol) to make a 1% solution of Victoria blue.
5. Add phenol to dye solution to a concentration of 1.5%

**Modified Victoria blue protocol.**

As for Victoria blue above but omit steps 2 and 3.

**Picro-Sirius red**

1. De-wax and hydrate paraffin sections.
2. Stain in picro-Sirius red for 30 mins.
3. Physically remove most of the dye from the slides by shaking, do not allow to dry out.
4. Quickly dehydrate in three changes of 100% ethanol. 
5. Clear in xylene and mount in DPX

Picro-sirius Red Solution:

0.5g Sirius red F3B (C.I. 35782) Direct Red 80

500ml Saturated aqueous solution of picric acid

**IHC-Elastin**

1. Take FFPE sections through xylene and alcohol to distilled water.
2. Incubate in 0.5% Trypsin (MP Biomedical)/ 0.5% Chymotrypsin / 1% Calcium Chloride (BDH) in Tris buffered saline pH 7.6 (TBS) for 30 minutes at 37 °C.
3. Soak slides in TBS with 0.04% Tween-20 for 5 minutes.
4. Block in peroxidase blocking solution for 5 minutes (Novolink^Tm^ Kit - Leica).
5. Wash in TBS for 5 minutes and blocked with protein block (Novolink^Tm^ Kit - Leica) for 5 mins.
6. Incubate for 1 hour at room temperature in elastin primary antibody (ab9519) at 1:100 in TBS.
7. Wash in TBS Tween 5 mins.
8. Incubate for 25 minutes in post primary (Novolink^Tm^ Kit - Leica), wash in TBS Tween 5 mins,
9. then 25 minutes in polymer solution (Novolink^Tm^ Kit - Leica).
10. Wash in TBS Tween 5 mins.
11. Develop with 3,3′ di-amino-benzidine (Novolink^Tm^ Kit - Leica).
12. Wash in TBS Tween 5 mins.
13. Counter stain in Mayer with Haematoxylin (Novolink^Tm^ Kit - Leica).
14. Dehydrate clear and mount.

**IHC multiplex Collagen I and Collagen III**

1. Take FFPE sections through xylene and alcohol to distilled water.
2. Incubate in 0.5% Trypsin (MP Biomedical)/ 0.5% Chymotrypsin (Sigma)/ 1% Calcium Chloride (BDH) in Tris buffered saline pH 7.6 (TBS) for 30 minutes at 37 °C.
3. Soak slides in TBS with 0.04% Tween-20 for 5 minutes.
4. Block in peroxidase blocking solution for 5 minutes (Novolink^Tm^ Kit - Leica).
5. Wash in TBS for 5 minutes and blocked with protein block (Novolink^Tm^ Kit- Leica) for 5 mins.
6. Incubate for 1 hour at room temperature in collagen III primary antibody (ab7778 - Abcam) at 1:300 in TBS.
7. Wash in TBS Tween 5 mins.
8. Incubate for 25 minutes in post primary (Novolink^Tm^ Kit - Leica), wash in TBS Tween 5 mins then 25 minutes in polymer solution (Novolink^Tm^ Kit - Leica).
9. Wash in TBS Tween 5 mins.
10. Develop with 3,3′ di-amino-benzidine (Novolink^Tm^ Kit - Leica).
11. Wash in TBS Tween 5 mins.
12. Place in 1L of pH 6.0 citrate buffer and microwave for 15 mins at 640W.
13. Soak slides in TBS with 0.04% Tween-20 for 5 minutes and block in peroxidase blocking solution for 5 minutes (Novolink^Tm^ Kit- Leica).
14. Block in alkaline phosphatase blocking solution for 5 minutes (Bloxall® - Vector Laboratories).
15. Wash in TBS for 5 minutes and block with normal horse serum (MP5402 – Vector Laboratories) for 5 mins.
16. Incubate for 1 hour at room temperature in collagen I primary antibody (ab6308 - Abcam) at 1:200 in TBS.
17. Wash in TBS Tween 5 mins.
18. Incubate for 25 minutes in Impress anti-mouse AP reagent (MP5402 – Vector Laboratories).
19. Wash in TBS Tween 5 mins.
20. Develop with fast red substrate (ab64254 - Abcam).
21. Wash in TBS Tween 5 mins then rinse in distilled water.
22. Allow to airdry to dehydrate, clear in xylene and mount.

**IHC-Collagen I**

1. Take FFPE sections through xylene and alcohol to distilled water.
2. Incubate in 0.5% Trypsin (MP Biomedical)/ 0.5% Chymotrypsin / 1% Calcium Chloride (BDH) in Tris buffered saline pH 7.6 (TBS) for 30 minutes at 37 °C.
3. Soak slides in TBS with 0.04% Tween-20 for 5 minutes.
4. Block in peroxidase blocking solution for 5 minutes (Novolink^Tm^ Kit - Leica).
5. Wash in TBS for 5 minutes and blocked with protein block (Novolink^Tm^ Kit - Leica) for 5 mins.
6. Incubate for 1 hour at room temperature in collagen III primary antibody (ab7778 - Abcam) at 1:500 in TBS.
7. Wash in TBS Tween 5 mins.
8. Incubate for 25 minutes in post primary (Novolink^Tm^ Kit - Leica), wash in TBS Tween 5 mins then 25 minutes in polymer solution (Novolink^Tm^ Kit - Leica).
9. Wash in TBS Tween 5 mins.
10. Develop with 3,3′ di-amino-benzidine (Novolink^Tm^ Kit - Leica).
11. Wash in TBS Tween 5 mins.
12. Counter stain in Mayer with Haematoxylin (Novolink^Tm^ Kit - Leica).
13. Dehydrate clear and mount.

**IHC multiplex Elastin and CK19**

1. Take FFPE sections through xylene and alcohol to distilled water.
2. Incubate in 0.5% Trypsin (MP Biomedical)/ 0.5% Chymotrypsin / 1% Calcium Chloride (BDH) in Tris buffered saline pH 7.6 (TBS) for 30 minutes at 37 °C.
3. Soak slides in TBS with 0.04% Tween-20 for 5 minutes.
4. Block in peroxidase blocking solution for 5 minutes (Novolink^Tm^ Kit - Leica).
5. Wash in TBS for 5 minutes and blocked with protein block (Novolink^Tm^ Kit - Leica) for 5 mins.
6. Incubate for 1 hour at room temperature in elastin primary antibody (ab9519) at 1:100 in TBS.
7. Wash in TBS Tween 5 mins.
8. Incubate for 25 minutes in post primary (Novolink^Tm^ Kit - Leica), wash in TBS Tween 5 mins then 25 minutes in polymer solution (Novolink^Tm^ Kit - Leica).
9. Wash in TBS Tween 5 mins.
10. Develop with 3,3′ di-amino-benzidine (Novolink^Tm^ Kit - Leica).
11. Wash in TBS Tween 5 mins.
12. Place in 1L of pH 6.0 citrate buffer and microwave for 15 mins at 640W.
13. Soak slides in TBS with 0.04% Tween-20 for 5 minutes and block in peroxidase blocking solution for 5 minutes (Novolink^Tm^ Kit- Leica).
14. Block in alkaline phosphatase blocking solution for 5 minutes (Bloxall® - Vector Laboratories).
15. Wash in TBS for 5 minutes and block with normal horse serum (MP5402 – Vector Laboratories) for 5 mins.
16. Incubate for 1 hour at room temperature in collagen I primary antibody (NCL-CK19 - Leica) at 1:100 in TBS.
17. Wash in TBS Tween 5 mins.
18. Incubate for 25 minutes in Impress anti-mouse AP reagent (MP5402 – Vector Laboratories).
19. Wash in TBS Tween 5 mins.
20. Develop with fast red substrate (ab64254 - Abcam).
21. Wash in TBS Tween 5 mins.
22. Counter stain in Mayer with Haematoxylin (Novolink^Tm^ Kit - Leica).
23. Wash in TBS Tween 5 mins then rinse in distilled water.
24. Allow to airdry to dehydrate, clear in xylene and mount.
